# Supplementary material for: Changes in the Fruit Quality Parameters of Medlar Fruit (Mespilus germanica L.) after Heat Treatment, Storage, Freezing or Hoarfrost
Source: Foods. 2023 Aug 16;12(16):3077. doi: 10.3390/foods12163077 (PMC10453525; doi:10.3390/foods12163077)
Supplement: Supplementary file 1 [file foods-12-03077-s001.zip › foods-2533301-supplementary.pdf]

Table S1. Identification and mean (± standard error) content (mg/kg FW) (n=6) of phenolic compounds in fresh medlar fruits at technological maturity in negative ions with HPLC-MS, MS^2 and MS^3.

Peak No.*	Phenolic compound	[M-H]⁻ (m/z)	MS^2 (m/z)	MS^3 (m/z)	Mean content ± SE (n=6)
	PHENOLIC ACIDS				532.85 ± 47.16
1	Protocatehuic acid	153	109		2.14 ± 0.19
2	3-Caffeoylquinic acid	353	191, 179, 135	173, 127, 85	473.74 ± 41.66
7	4-Caffeoylquinic acid	353	173, 179, 191		11.93 ± 1.13
6	5-Caffeoylquinic acid	353	191, 179, 173, 135		19.81 ± 2.62
4	Caffeic acid hexoside	341	179		11.90 ± 1.29
5	Sinapic acid	223	208, 179, 164	164, 149, 135	0.65 ± 0.06
5	3-*p*-Coumaroylquinic acid	337	163		5.44 ± 0.51
11	4-*p*-Coumaroylquinic acid	337	173, 163, 155, 137, 191		0.77 ± 0.118
13	5-*p*-Coumaroylquinic acid 1	337	191, 173, 163, 155	127, 93, 85	1.40 ± 0.24
15	5-*p*-Coumaroylquinic acid 2	337	191, 173, 163,		1.01 0.203
7	3-Feruloylquinic acid 1	367	193,134	149	1.07 ± 0.101
9	5-Feruloylquinic acid 2	367	191	173, 127, 85	0.54 ± 0.050
22	Ellagic acid	301	271, 257, 229, 185		2.46 ± 0.35
	FLAVANOLS				375.21 ± 45.71
5	Catechin	289	245, 205, 179, 261	203, 227	0.72 ± 0.0066
9	Epicatechin	289	245, 205, 179, 261	203, 227, 161	38.59 ± 3.60
7	Procyanidin dimer 1	577	451,425,407,289		85.06 ± 8.08
3	Procyanidin trimer 1	865	577, 451, 425, 407, 289		41.58 ± 4.24
10	Procyanidin trimer 2	865	577, 451, 425, 407, 289		30.74 ± 3.30
14	Procyanidin trimer 3	865	577, 451, 425, 407, 289		12.77 ± 2.31
16	Procyanidin trimer 4	865	577, 451, 425, 407, 289		20.78 ± 4.70
8	Procyanidin tetramer 1	1153	865,803,425,451,559,289		92.23 ± 14.71
12	Procyanidin tetramer 2	1153	865,863,425,451,559,287		52.74 ± 8.06
	FLAVONES				1.48 ± 0.314
26	Apigenin hydroxyhexoside	449	269	225, 183, 151	1.48 ± 0.314
	FLAVANONES				1.65 ± 0.275
17	Naringenin hexoside 1	433	271	151	0.968 ± 0.104

18	Naringenin hexoside 2	433	271	151	0.321 ± 0.080
21	Naringenin hexoside 3	433	271	151	0.36 ± 0.101
	FLAVONOLS				16.84 ± 0.381
19	Quercetin-3-galactoside	463	301	179, 151	0.784 ± 0.116
20	Quercetin-3-glucoside	463	301	179, 151	0.722 ± 0.08
18	Quercetin-3-rutinoside	609	301	179, 151	0.558 ± 0.139
23	Quercetin-pentoside	433	301	179	0.17 ± 0.017
17	Quercetin dirhamnoside	593	447	301	0.484 ± 0.052
23	Quercetin-rhamnosyl hexoside	609	463	301	1.01 ± 0.099
17	Laricitrin rhamnoside	477	331		0.807 ± 0.087
20	Kaempferol hexoside 1	447	285	257, 229	0.031 ± 0.004
25	Kaempferol hexoside 2	447	285	257, 229	0.322 ± 0.044
27	Dihydrokaempferol hexoside	449	287		11.44 ± 1.38
18	Isorhamnetin hexoside 1	477	315	300, 273	0.147 ± 0.036
24	Isorhamnetin hexoside 2	477	315	300, 273	0.359 ± 0.068

*Peak No….Peak number on the chromatogram (Figure 1).
